# *MTHFR C677T* mutation affects adipogenic differentiation abilities of human bone marrow-derived mesenchymal stem cells

**DOI:** 10.55730/1300-0152.2624

**Published:** 2022-07-14

**Authors:** Rengim VURAL, Dicle ÇELİK, Ersin Berkay PEKER, Ekin KÖNİ, Ayşe Aydanur KULAÇ, Zeynep TOKCAER KESKİN

**Affiliations:** 1 Department of Molecular and Translational Biomedicine, Graduate School of Natural and Applied Sciences, Acıbadem Mehmet Ali Aydınlar University, İstanbul, Turkey; 2 Department of Molecular Biology and Genetics, Faculty of Engineering and Natural Sciences, Mehmet Ali Aydınlar University, İstanbul, Turkey; 3 Acıbadem Labcell, İstanbul, Turkey

**Keywords:** 5,10-Methyltetrahydrofolate reductase (*MTHFR*), human bone marrow-derived mesenchymal stem cells (hBM-MSCs), C677T, polymorphism, adipocyte, differentiation

## Abstract

The effects of *5,10-Methylenetetrahydrofolate reductase* (*MTHFR*) C677T polymorphism on human bone marrow-derived mesenchymal stem cells (hBM-MSCs) viability, morphology, physiology and differentiation capacity were investigated in this study. For this purpose, primary hBM-MSCs with wild type (WT, C/C), heterozygote (HTZ, C/T) and homozygote (HMZ, T/T) for the *MTHFR* gene were obtained with ethical committee permission and donor informed. Mutations were detected using RFLP and Sanger sequencing methods from genomic DNA isolated from cells, colonization properties were investigated by CFU-F test and proliferative differences were investigated by MTT test. Adipogenic, osteogenic, and chondrogenic differentiation were induced to study changes in their differentiation potentials, and the results were statistically analyzed using one-way ANOVA with Graphpad Prism. A total of 13 donors were screened and there were no differences in the hBM-MSC markers and in vitro morphologies of the cells. While there were significant differences between WT and HTZ as a result of the CFU-F test, there were no significant differences in the MTT test after 24 and 48 h. As a result of differentiation tests, it was found that adipogenic differentiation was significantly more in HMZ cells than WT cells. Osteogenic and chondrogenic differentiation results did not give statistically significant results. As a result of these experiments, adipogenic differentiation was found to be affected by the *MTHFR* genotype in hBM-MSCs.

## 1. Introduction

5,10-Methyltetrahydrofolate reductase (*MTHFR*) enzyme is involved in folate metabolism, which regulates DNA, RNA and protein methylation of cell metabolism. *MTHFR* converts 5,10-methyltetrahydrate to 5-methyltetrahydrate. 5-methyltetrahydrofolate produced in this way acts as a methyl donor for the conversion of homocysteine to methionine (Goyette et al., 1998). This transformation is catalyzed by the enzyme methionine synthase found in all mammalian tissues and vitamin B12 is used as a cofactor (Günebak Şahin ÇT, 2012). Therefore, all mutations in the *MTHFR* gene that alter enzyme function cause deterioration in the pathways of methylation and DNA synthesis. The most common mutation in the *MTHFR* gene is C677T polymorphism. This polymorphism is located in exon 4 and results in the conversion of Valin amino acid to Alanine amino acid in the codon 222 region. C677T polymorphism alters the thermostability of the enzyme produced, causing its activity to decrease (Rosenberg et al., 2002).

According to the literature, *MTHFR* C677T polymorphism has been associated with many different types of diseases. There are cardiovascular diseases such as stroke (Banerjee et al., 2007), coronary artery diseases (Verhoef et al., 2002), infertility and pregnancy loss (Cao et al., 2013; Rai and Kumar, 2017), down syndrome (Rai et al., 2014), diseases associated with pregnancy such as Spina Bifida (Shaw et al., 1999), neurological and psychiatric problems such as Parkinson’s, Alzheimer’s, schizophrenia (Hua et al., 2011; Wu et al., 2013; Hu et al., 2015), diabetic neuropathy (Yang et al., 2013), many different types of cancer (Ergul et al., 2003; Izmirli et al., 2011; Küçükhüseyin et al., 2011; López-cortés et al., 2013; Ozen et al., 2014; Xu et al., 2013), bone-related diseases such as osteoporosis (Villadsen et al., 2005) and autoimmune diseases such as Behçet disease (Özkul et al., 2005). The common presence of C677T polymorphism in the *MTHFR* gene in all these different types of diseases demonstrates the importance of this gene and related mutation. Christensen et al. (1997) reported in animal studies that this enzyme can show the activity of around 40% in homozygous individuals, and around 60% in heterozygous.

Mesenchymal stem cells (MSCs) are adult stem cell types whose therapeutic use in regenerative medicine and tissue engineering is quite common and valuable. These cells have the potential to transform into many different cell types such as osteocyte (bone cell), adipocyte (fat cell), and chondrocyte (cartilage cell) (Kolios and Moodley, 2012). Although MSCs can be isolated from many different regions such as adipose tissue, amniotic fluid, peripheral blood, the most commonly used type of mesenchymal cells is isolated from bone marrow (bone marrow-derived mesenchymal stem cells, BM-MSCs) (Ding et al., 2011).

Because of *MTHFR* C677T polymorphism, homocysteine levels increase. High homocysteine level is considered as an indicator for bone and heart diseases, and trigger the formation of osteoclasts, causing a decrease in bone density (Behera et al., 2017). Another study has shown that high concentrations of homocysteine cause apoptosis in BM-MSCs (Cai et al., 2013). Behçet’s disease is also associated with *MTHFR* C677T polymorphism. Davatchi et al. (2013) studied the regenerative capacities of autologous MSCs in the treatment of vasculitis occurring due to Behçet’s disease and it was examined to see if there was a change in their eyesight. However, this study, which was carried on three patients, it was concluded that there was no improvement in their eyesight and that stem cell therapy was unsuccessful (Davatchi et al., 2013). In another Behçet disease study, the significant decrease in central nervous system damage was observed when allogeneic MSCs were given to a patient with central nervous system damage (Marmont et al., 2006). The most important difference that stands out in these two studies is the genetic infrastructure of the MSCs used. This suggests that genetic changes that are effective in Behçet’s disease may be acting upon the differentiation and regeneration capacity of MSCs.

MSCs can be transferred to the body, in regenerative applications for the cellular treatment, either by autologous or allogeneic transplantation in order to repair the damaged tissue. This research study aimed to investigate the effects of *MTHFR* C677T polymorphism on the differentiation capacities, vitality, clonality and morphology of hBM-MSCs obtained from real donors and according to our results, *MTHFR* C677T homozygous polymorphism affects the CFU-F and adipogenic differentiation capacities of hBM-MSCs.

## 2. Materials and methods

### 2.1. Cells and their maintenance

Primary hBM-MSCs were obtained from Acıbadem Labcell and used in this research study with ethical approval of Acıbadem Mehmet Ali Aydınlar University ethics committee, with ATADEK 2019/1-13 approval number.

hBM-MSCs were plated on cell culture plates covered with 0.1% Gelatin (Sigma, 9391) and cultured in MSC Nutristem XF medium (Biological Industries, 05-200-1A) supplemented with MSC Nutristem XF Supplement, in 37 °C, 5% CO2 incubator. The medium was changed every 2–3 days till the cells reached 80% confluency and passaged 1:4 by using 0.05% 1X Trypsin EDTA (Gibco, 25300054).

### 2.2. DNA isolation

For DNA isolation, 1 × 10^6^ of BM-MSC were incubated in 300 uL lysis buffer containing 240 ug proteinase K at 55 °C for 1.5 h by agitation. One hundred microliters of 5M NaCl solution was added and kept on ice for 10 min. Samples were centrifuged at 4 °C, 3000 rpm for 30 min and the supernatant is treated with 800 uL of isopropanol at RT for 15 min. Tubes were centrifuged at RT with 13,000 rpm for 10 min. One milliliter of 70% EtOH was added onto the pellet and the tubes were washed by inverting gently. After washing, it was centrifuged at RT and maximum rpm for 10 min. EtOH was removed from the tubes and then they were kept upside down until EtOH was completely evaporated. DNA samples were dissolved in 100 uL of ddH2O and their concentration was determined by Nanodrop.

### 2.3. Mutation determination

Restriction fragment length polymorphism (RFLP) assay was performed in order to determine C>T mutation on *MTHFR* gene. Primers that amplified for all genes were designed by using Primer Blast (NCBI) (Table 1.1). A total of 100 ng of DNA obtained from BM-MSCs, the 389 bp region surrounding the mutation were amplified by polymerase chain reaction (PCR) (T100 Thermal Cycler, BioRad, 1801096) (Tables 1.2 and 1.3). The C>T mutation creates a recognition site for the *Hinf*I restriction enzyme. Since there is a *Hinf1* recognition sequence in the amplified region of the *BNP* gene, it was used as a positive control in all reactions. The amplified region was cleaved by *Hinf*I enzyme for 1 h at 37 °C (T100 Thermal Cycler, BioRad, 1801096) (Table 1.4). The expectation for the enzyme cleavage was a single band (389bp) in wild type (WT) BM-MSCs which carry no mutation, two bands (173 bp and 217 bp) in the homozygous mutant (HMZ) BM-MSCs and three bands (389 bp, 173bp and 217 bp) in heterozygous mutant (HTZ) BM-MSCs.

Following the RFLP assay, PCR amplicons were also subjected to Sanger sequencing, simultaneously in order to confirm the results. Sequencing was performed by MCLAB (Molecular Cloning Laboratories, ABD) (https://www.mclab.com/DNA-Sequencing-Services.html) using the *MTHFR* forward primer, and the sequencing result was analyzed with CLC Main Workbench 8 software.

### 2.4. Determination of cell proliferation rate by MTT assay

Assessing metabolic activity of BM-MSCs of each donor was performed with triplicates and a control group. The BM-MSCs (WT, HMZ and HTZ) were seeded into 1% gelatin-coated F bottom 96-well with 10 × 10^3^ cells/ well. The incubation has been started for 24 h and 48 h at the same time in a humidified atmosphere with at 5% CO_2_ at 37 °C. Ten microliters of MTT formazan dye, which was used to indicate viable cells, was added into each well after the incubation period. Four hours later, 100 uL of solubilization buffer was added into each well to dissolve insoluble formazan crystals and incubated in the dark overnight. Dissolved formazan crystals provided a colored solution for quantification by measuring the absorbance. The absorbance values were obtained by Varioskan instrument readouts and the results were statistically analyzed with GraphPad Prism.

### 2.5. Investigating the colonization property by colony forming unit-fibroblast assay

Colony-forming unit-fibroblast (CFU-F) assay has been set up by coating the 6-well cell culture plates with 0.1% gelatin (Sigma, 9391) and seeding the BM-MSCs of the donors as to be 40 × 10^3^ cells/well. The plates were prepared as duplicates for all WT, HMZ and HTZ groups. The mix of MSC Nutristem XF medium (Biological Industries, 05-200-1A) and MSC Nutristem XF Supplement was freshened in every 3 days as prewarmed before using for 10 days. For colony detection, the colonies were stained with Giemsa stain stock solution (Sigma G5637-5G) diluted with 1X DPBS in 1:10 ratio. The cells in each well were washed with 1X DPBS twice. Ice cold 99.9% methanol (0 °C) was added for fixation for 10 min. After incubation time is over, methanol was aspirated and the plates were left uncovered to let the remaining methanol to evaporate. Giemsa working solution (1:10 diluted stock solution) was added at a volume of 2 mL to cover the surface of the cells in each well and incubated for 10 min at RT. The wells were washed with plenty of ddH_2_O for 3 times until all the dye residues were removed. For the quantitation, the wells were imaged to calculate colony numbers and sizes by using ImageJ (v. 1.53a) and the results were statistically analyzed with GraphPad Prism.

### 2.6. Differentiation of BM-MSCs

#### 2.6.1. Adipocyte differentiation of BM-MSCs

For the adipocyte differentiation, 6-well plates were prepared by coating with 0.1% gelatin. The cells counted with trypan blue solution were seeded into wells at a density of 10 × 10^4^ cells/well. The experimental set-up was established with duplicates for each donor and one control group which did not receive differentiation medium. The cells were incubated in 5% CO_2_ incubator at 37 °C until they reach at least 80% confluency, with the renewal of the prewarmed MSC medium in every 2–3 days. The combined StemPro Adipogenesis Differentiation Basal Medium (PromoCell, C-28016) and StemPro Adipogenesis Supplement (PromoCell, C-39816) were used to initiate the adipogenesis of the cells reached the desired confluency. The cells were cultured for 12 days and their medium was changed with a fresh medium once in every 3 days. At the end of the differentiation period, the medium was aspirated and washed with 1X PBS, then fixed with 4% paraformaldehyde solution for 30 min at RT. The fixation was ended by washing them with 1X PBS twice and with ddH_2_O 3 times. After the washing process, the cells were covered with OilRedO (Sigma O0625-25G) solution in order to stain lipid droplets in differentiated cells for 50 min at RT. The cells were washed with ddH_2_O 3 times to remove the dye residues. In the end, the cells were kept with ddH2O in order to prevent them from drying until their images were captured. For the photos, Zeiss AX10 microscope (Carl Zeiss, USA) was used. ImageJ (v. 1.53a), the image processing program, was used for quantitation based on stained lipid droplets of adipogenesis from the collected photos.

#### 2.6.2. Osteocyte differentiation of BM-MSCs

For the osteocyte differentiation, 6-well plates were prepared by coating with 0.1% gelatin. The cells counted with trypan blue solution were seeded into wells at a density of 10 × 10^4^ cells/well. The experimental setup was established with duplicates for each donor and one control group which did not receive differentiation medium. The cells were incubated in 5% CO_2_ incubator at 37 °C until they reach at least 80% confluency, with the renewal of the prewarmed MSC medium in every 2–3 days. The combined StemPro Osteocyte Differentiation Basal Medium (PromoCell, C-28013) and StemPro Osteogenesis Supplement (PromoCell, C-39813) were used to initiate the osteogenesis of the cells reached the desired confluency. The cells were cultured for 12 days and their medium was changed with a fresh medium once in every 3 days. At the end of the differentiation period, the medium was aspirated and washed with 1X PBS, then fixed with 70% ethanol for 1h at RT. Fixation was ended by washing the cells with ddH_2_O twice. After the washing process, the cells were covered with Alizarin Red-S (Merck 1062780025, pH 4,1-4,3) in order to stain calcium deposits on differentiated cells for 30 min at RT in dark. Unbound dye was washed with ddH_2_O 3 times. The stained cells were kept with ddH2O in order to prevent them from drying until their images were captured with Zeiss AX10 light microscope (Carl Zeiss, USA). ImageJ (v. 1.53a) was used for quantitation based on the stained calcium deposits of osteogenesis from the collected photos.

#### 2.6.3. Chondrogenesis differentiation of BM-MSCs

Before the initiation of chondrogenesis differentiation, round-bottom (U bottomed noncoated and nonadherent) 96-well plates were used in order to facilitate sphere formation at the density of 15 × 10^4^ BM-MSCs during the 48-h incubation in 5% CO_2_ incubator at 37 °C. Chondrogenesis has been started with the combined StemPro Chondrocyte Differentiation Basal Medium (PromoCell, C-28012) and StemPro Chondrogenesis Supplement (PromoCell, C-39812) with replacing with the fresh prewarmed differentiation medium once in 3 days. There were duplicates and a control group, which was replaced with prewarmed MSC medium, used as the experimental set-up and the process ended up after the 21 days period. All the spheres were washed with 1X PBS twice in order to remove the medium and fixed with 10% formalin for 60 min at RT. The fixed spheres were washed with ddH_2_O twice and stained with Alcian Blue (Sigma, TMS-010-C, pH 2.5) overnight at RT in a dark place, covered with aluminum foil. To remove excess dye, destain solution was added into each well for 10 min twice and then the spheres were washed with 1X DPBS. The chondrogenic differentiation was demonstrated by capturing images of the spheres. Semiquantification was also performed to indicate the experimental results.

Semiquantification was based on the Alcian blue elution from the spheres. The absorbance measurement via Varioskan readout of eluted Alcian blue demonstrated the differentiation level of the cells. 8M Guanidine HCl solution (GuHCl) was prepared to elute Alcian blue from the spheres. The 1X PBS was removed and 150 uL/well of 8M GuHCl was added into each well and incubated overnight at 2–8 °C. The absorbance was read at 600 nm. There were also control groups within the wells containing only 8M GuHCl.

### 2.7. Statistical analysis

The data was indicated as the means +/− SD and analyzed by GraphPad Prism (GraphPad Software, La Jolla, CA, USA). One-way ANOVA with Tukey’s multiple comparisons test (between multiple groups) was used to analyze and p < 0.05 was considered significant.

## 3. Results

### 3.1. Genotyping the C667T polymorphism

**Figure 1:**
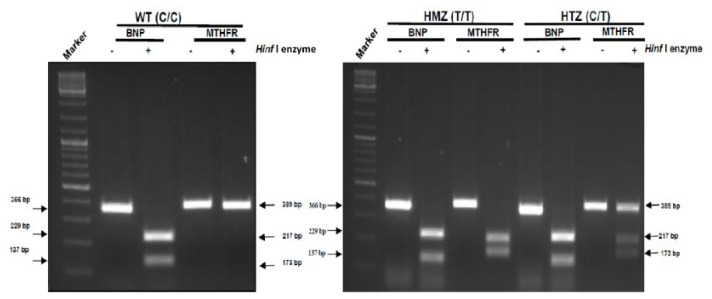
Genotyping of BM-MSCs by RFLP assay. The demonstration of WT, HMZ and HTZ genotypes (respectively) by the size of bands.

All BM-MSCs were genotyped by using restriction fragment length polymorphism (RFLP) assay. The enzyme cleavage of BM-MSC donors has been given different sizes as base pairs. The wild type (WT) donor without mutation was demonstrated with a single band at 389 bp. The homozygous mutated (HMZ) donors resulted in 2 bands with the size of 173 bp and 217 bp. Finally, the heterozygous mutated (HTZ) donors resulted in 3 bands with the size of 389 bp, 217 bp and 173 bp. The human *BNP* gene PCR product was used as a positive control in all reactions according to its *Hinf1* recognition site in the amplified region. The *BNP* product gives a single band with the size of 366 bp. *Hinf1* enzyme digests *BNP* PCR product into 2 sequences with 229 bp and 137 bp (Figure 1). For each genotype, one representative run was demonstrated in RFLP results.

RFLP results were confirmed by performing Sanger sequencing and sequencing results were analyzed by CLC Workbench 8. *MTHFR* gene is on (−) strand of the genomic DNA and the polymorphism we searched for is also referred to as rs1801133. The samples were compared with the NG_013351 coded genomic DNA sequence, obtained from the NCBI genome browser. Since we used a forward primer for sequencing, the mutation corresponded to the 218th nucleotide in the sequence by indicating the SNP C677T as G >A change.

**Figure 2:**
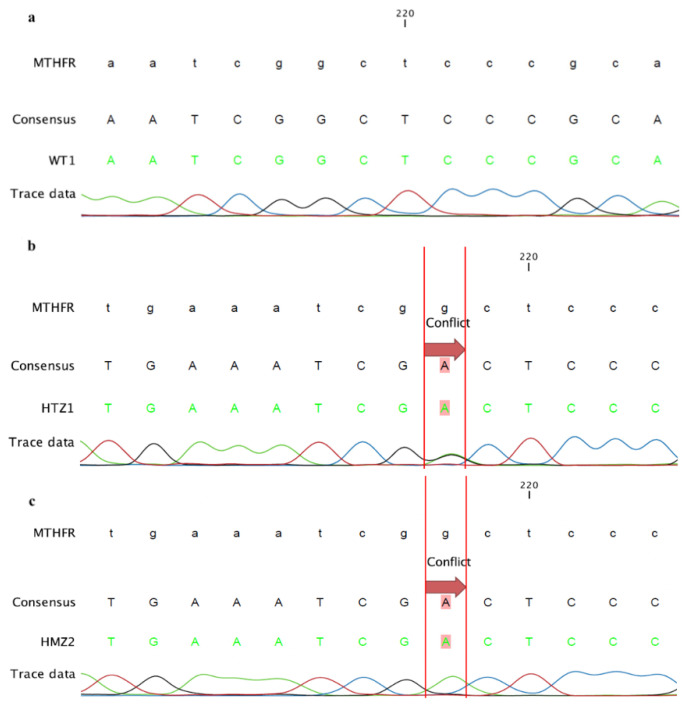
Representative Sanger sequencing results of the BM-MSCs. **a)** The representation of assembly of the WT 1**. b)** The representation of assembly of the HTZ 1. **c)** The representation of assembly of the HMZ 2.

For the wild type donors when tracer data is followed, it has been seen that G nucleotide is on the 218th base. Since both alleles have G nucleotides, there is only one trace data represented in black (Figure 2a).

For the heterozygous donors, the tracer data demonstrates that there is A nucleotide as well as G nucleotide is at the 218th base. Since both alleles have 2 different bases, there are two trace data represented by black and green color which belong to the HTZ donors (Figure 2b).

For the homozygous donors, the tracer data demonstrates the G nucleotide is replaced by nucleotide A at the 218th base. Both alleles have A nucleotide, there is one trace represents the green color (Figure 2c).

Among all the samples sequenced 3 WT, 3 HTZ, and 3HMZ were used in further experiments and one representative tracer data was demonstrated in Figure 2.

### 3.2. Characterization of human BM-MSCs by flow cytometry

**Figure 3:**
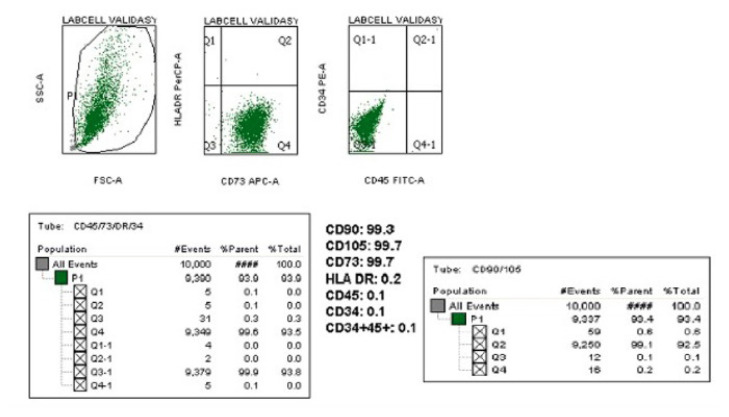
Representative flow cytometry image of BM-MSC surface marker characterization.

The human BM-MSCs used in this study were previously isolated and characterized by Acıbadem Labcell (Acıbadem Health Group Co.). The characterization was performed by using flow cytometry based on MSC markers validated by ISCT (Dominici et al., 2006).The flow cytometry results indicated that all the BM-MSCs obtained from all donors expressed the MSC markers over 95% which are CD73, CD90 and CD105. In addition to that, as a negative demonstration, the hematopoietic markers were also investigated. It was observed that the hematopoietic markers, HLA-DR, CD34 and CD45, were also not expressed by any of the hBM-MSCs. In all BM-MSCs, the negative marker expressions were below 1% (Figure 3, Table 2).

### 3.3. The effect of *MTHFR C677T* polymorphism on BM-MSC morphology

**Figure 4:**
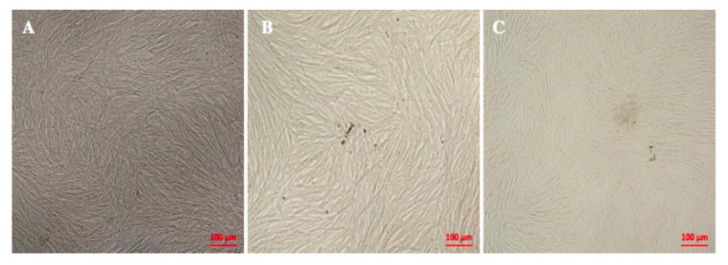
The morphology of BM-MSC donors under the light microscope. **a)** Wild-type (WT). **b)** Heterozygous (HTZ). **c)** Homozygous (HMZ).

The cells that evaluated for the morphological and proliferative differences were at the passage number (P) between 4 and 7. *MTHFR* C677T polymorphism did not show any significant difference between the donors when heterozygous and homozygous donors compared to wild type donor under the light microscope (Figure 4). That is why we wanted to further evaluate the effect of *MTHFR* C677T SNP in their proliferation potentials.

### 3.4. The effect of *MTHFR C677T* polymorphism on BM-MSC proliferation

**Figure 5:**
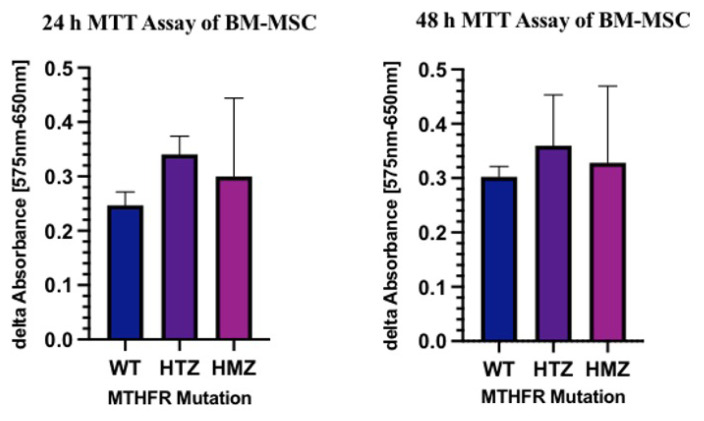
MTT assay results of BM-MSCs for 24 h and 48 h.

The MTT assay was applied to all 3 biological replicates from each genotype and 3 technical replicates from each sample for both 24 h and 48 h with 10,000 cells/well in 96-well plates. The MTT results were plotted with GraphPad Prism and no significant difference was found (Figure 5) (p values for 24 h and 48 h are 0.4597 and 0.7824, respectively). The comparative 24-h p values between WT-HMZ, WT-HTZ, HTZ-HMZ were 0.8619, 0.5475, 0.9271, and 48-h p values were 0.9860, 0.8765, 0.9751, respectively. The analysis was performed by using Tukey’s multiple comparison test between allelic groups.

### 3.5. Evaluating the effect of *MTHFR C677T* polymorphism on BM-MSCs by colony-forming unit-fibroblast (CFU-F) assay

**Figure 6:**
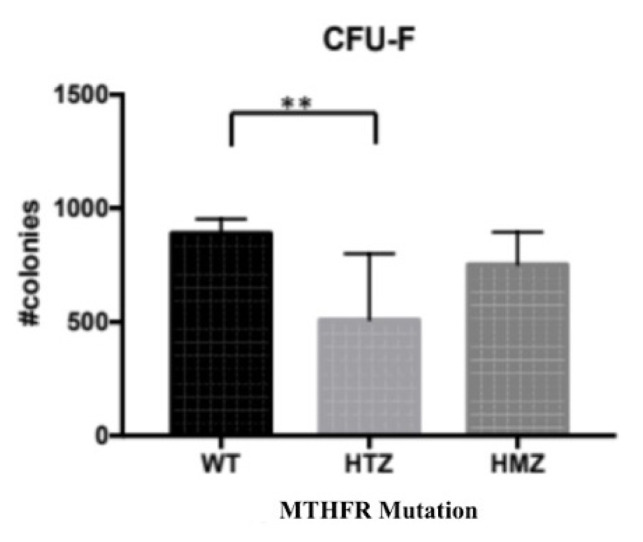
Relation between *MTHFR* mutation and CFU of BM-MSCs. As a result of quantitation by ordinary one-way ANOVA test (GraphPad Prism), the comparison of colony numbers showed a significant difference between WT-HTZ genotypes (p = 0.093) (**). p values of comparison of WT-HMZ and HTZ-HMZ are 0.4430 and 0.1024, respectively (p < 0.05).

Colony-forming unit-fibroblast (CFU-F) assay is one of the main characterizations of MSCs. the cells were seeded as 40 × 10^3^ cells/ well in 6-well plates and cultured for 10 days. The images of Giemsa stained colonies were analyzed by using ImageJ and a significant difference between WT-HTZ CFU numbers was revealed by using GraphPad Prism (p = 0.093) (Figure 6). However, there was no significant difference between HTZ-HMZ and WT-HMZ (p values were 0.4430 and 0.1024, respectively). The analysis was performed by using Tukey’s multiple comparison test between allelic groups.

### 3.6. The effect of *MTHFR C677T* polymorphism on adipogenic differentiation of BM-MSCs

**Figure 7:**
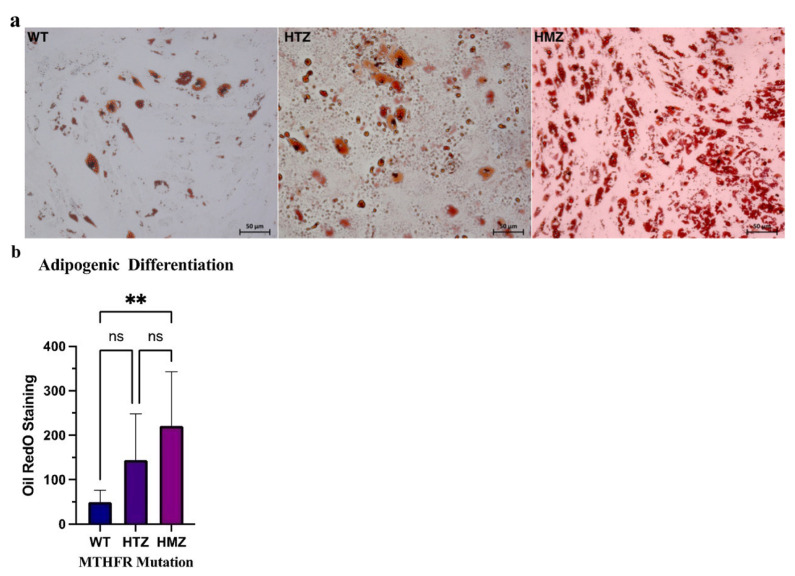
The results of adipogenic differentiation assay. **a)** The light microscopy images of BM-MSCs after adipogenic differentiation stained with Oil Red O (WT, HTZ and HMZ, respectively). **b)** Quantification of adipogenic differentiation assay by one-way ANOVA with Tukey’s multiple comparison test. Significance difference between WT-HMZ genotypes (p = < 0.0010) (**) (p < 0.05). There was not any significant difference between WT-HTZ genotypes (p = 0.0795) and HTZ-HMZ (p = 0.1816).

Adipogenic differentiation experiments were visualized (Figure 7a) and quantified based on the presence of red lipid droplets in the staining area. As a result of quantification by using GraphPad Prism, there was a significant difference between WT-HMZ (p = < 0.0010) (**) genotypes while there was not any significant difference between WT-HTZ and HTZ-HMZ genotypes (p values were 0.0795 and 0.1816, respectively) (Figure 7b). The analysis was performed by using Tukey’s multiple comparison test between allelic groups.

### 3.7. The effect of *MTHFR C677T* polymorphism on osteogenic differentiation of BM-MSCs

**Figure 8:**
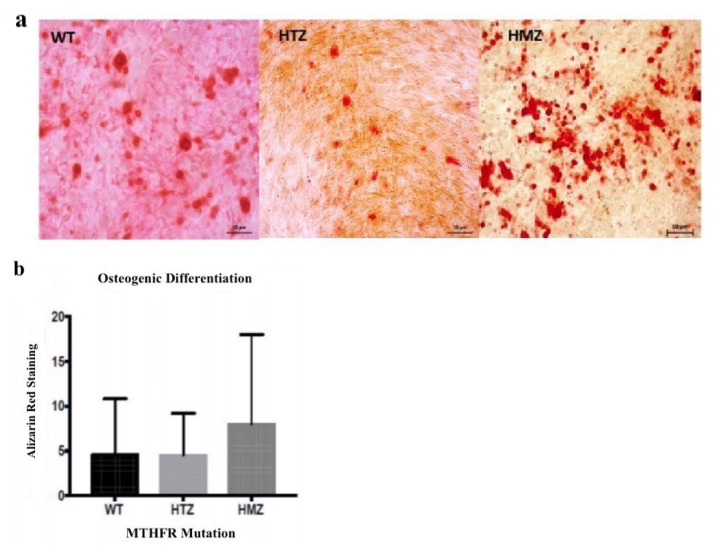
The results of osteogenic differentiation assay. **a)** The light microscopy images of BM-MSCs after adipogenic differentiation stained with Alizarin Red-S dye (WT, HTZ and HMZ, respectively). **b)** Quantification of osteogenic differentiation assay by one-way ANOVA with Tukey’s multiple comparison test. There was not any significant difference between WT-HTZ (p = 0.999), WT-HMZ (p = 0.7041) and HTZ-HMZ (p = 0.6952).

Osteogenic differentiation experiments were visualized (Figure 8a) and quantified based on the presence of calcium deposits in the staining area. As a result of quantification by using GraphPad Prism, there was no significant difference between WT-HMZ, WT-HTZ and HTZ-HMZ genotypes (Figure 8b). The analysis was performed by using Tukey’s multiple comparison test between allelic groups; WT-HTZ, WT-HMZ and HTZ-HMZ (p values were 0.9999, 0.7041 and 0.6952, respectively).

### 3.8. The effect of *MTHFR C677T* polymorphism on chondrogenic differentiation of BM-MSCs

**Figure 9:**
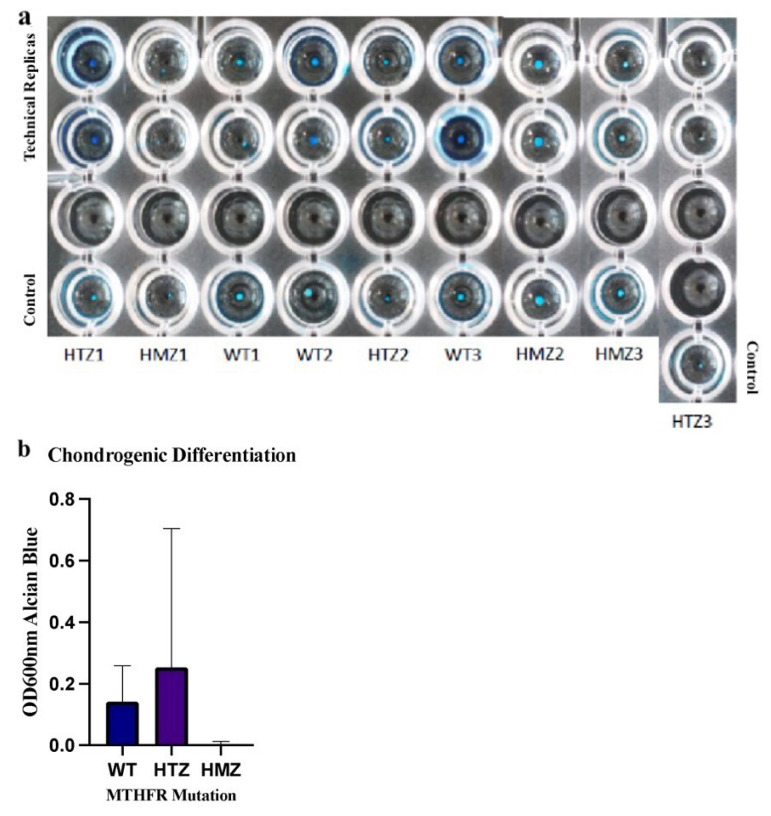
The results of osteogenic differentiation assay. **a)** The light microscopy images of BM-MSCs after adipogenic differentiation stained with Alcian blue dye. **b)** Quantification graph by using ordinary one-way ANOVA with Tukey’s multiple comparison test. There was no significant difference between WT-HTZ (p = 0.7313), WT-HMZ (0.5691) and HTZ-HMZ (p = 0.1620) genotypes.

Chondrogenic differentiation was visualized (Figure 9a) and all allelic groups have important differences between their biological replicates and there were differences between each other according to Alcian blue staining.

Semiquantification results, performed by measuring the OD600nm absorbance value, demonstrated that there was no significant difference between allelic groups (Figure 9b). The analysis was performed by using Tukey’s multiple comparison test and the comparative p values of WT-HTZ, WT-HMZ, and HTZ-HMZ are 0.7313, 0.5691, and 0.1620, respectively. However, it was also noticed that the HMZ groups were poorly differentiated into chondrocytes.

## 4. Discussion

In our study, we investigated the effect of *MTHFR* C677T polymorphism on BM-MSCs. We obtained remarkable results specifically for the effect of the polymorphism on the adipogenic differentiation potential of the cells. There are several studies demonstrated the underlying defects of the differentiation mechanism. It was reported that adipogenesis increased when folate metabolism was inhibited or S-adenomethionine inhibitors were used. It was deduced that the decrease in DNA methylation profile is the reason of increased adipogenesis (Gouffon, 2012). These kinds of defects in folate metabolism can be emerged by a mutation on the *MTHFR* gene like *MTHFR* C677T polymorphism. Di Renzo et al. (2014) reported that *MTHFR* C677T polymorphism was found to be directly proportional to the increase in body fat mass and decrease in muscle ratio, and they showed that obese patients with metabolic syndrome could lose weight rapidly with a hypocaloric diet. Despite all these studies, changes in differentiation cannot be explained by the *MTHFR* gene alone according to the report by (Nic-Can et al., 2019) which demonstrates the importance of epigenetic factors on differentiation potential of MSCs. Despite the differences in standard deviations, our findings also support the current findings in the literature.

*MTHFR* C677T polymorphism and its relation with bone related-diseases is known and this relation has been reported previously (Villadsen et al., 2005). High homocysteine (Hcy) levels due to *MTHFR* C677T polymorphism cause some disorders in the bone. For this reason, our negative findings in osteocyte differentiation can be explained by in vitro conditions, where no continuous homocysteine accumulation is present. *MTHFR* polymorphism, which causes long-term homocysteine induced damage in physiological conditions cannot be provided in vitro. As a result, the C677T mutation may not directly affect the osteogenic differentiation potential of BM-MSCs in our experimental setup.

The last investigation for the differentiation ability was the chondrogenic differentiation assay and there was no significant result. All allelic groups had important differences between their biological replicates. It was clearly observed that HMZ genotype replicates did not respond to the chondrogenic differentiation despite the experimental repeats. This can be explained according to the report demonstrated that the T allele frequency is increased in osteoarthritis patients with *MTHFR* C677T polymorphism compared to healthy individuals. It is also reported that the patients with osteoarthritis were observed 6 times more with homozygous TT genotype (Inanir et al., 2013). In another study, it is demonstrated that epigenetic regulation of the *WISP1* gene plays an essential role in cartilage degeneration in osteoarthritis patients. Both hyper- and hypomethylation of this gene take place in cartilage differentiation (van den Bosch et al., 2015). These two studies can be an explanation of our results. Our HMZ genotype replicates did not differentiate according to the possibility of inhibition of methylation due to *MTHFR* mutation. This may have caused the changes in the regulation of important genes in cartilage differentiation.

Runx2 is an important transcription factor that plays a role in both osteogenic and chondrogenic differentiation. A study reported that there are multiple functions of Runx2 in the regulation of chondrogenic differentiation. It plays a role in the maintenance of chondrocyte phenotype while inhibiting adipogenesis. In the study, they depleted the Runx2 and observed the loss of differentiated phenotype of chondrocytes (Enomoto et al., 2004). In our study even though we did not check the levels of Runx2, we observed a significant increase in HMZ adipogenic differentiation and almost no differentiation in chondrogenic differentiation setup. That is why the expression levels of Runx2 could be investigated in further experiments. It must also be considered that *MTHFR* gene might not be the only reason that affects the differentiation potential due to donors’ background genetic mutations.

When the CFU-F results were considered a significant difference between WT and HTZ allelic groups was obtained. This result can be concluded according to the essential role of *MTHFR* protein in folic acid metabolism and in methylation/remethylation cycles. With the C677T mutation, the protein gains a heat-labile structure and its activity decreases by 35% in heterozygous genotype and 50%–70% in homozygous genotype. In the presence of this mutation, proliferation or cellular morphology was not affected, but a significant decrease in HTZ genotype may be explained by a change in the balance of progenitor and stem cells. It is known that DNA methylation and demethylation processes play an important role in stem cell differentiation. On the other hand, in HMZ cells, since the methylation cycle is not sufficiently well established, spontaneous differentiation or the number of progenitor cells may be decreased and the number of stem cells may be more preserved. For this reason, there may not be a significant difference between WT-HMZ. In order to prove this inference, epigenome differences need to be investigated. In a study investigating the relationship between oral epithelial cells and *MTHFR* C677T mutation, the result was controversial although there was no difference in global methylation levels.

Finally, our results did not find any significant difference in the proliferative potential of the cells in the presence of *MTHFR* C677T mutation. The morphology and the CD marker signature of the cells were not different from the WT, but the cells demonstrated the most important difference at the adipogenic differentiation potential of BM-MSCs

We have investigated the effect of *MTHFR* C677T polymorphism on BM-MSCs because the *MTHFR* gene was related with a wide range of different diseases such as coronary artery and cardiovascular diseases, psoriasis, infertility, Parkinson’s disease, Alzheimer’s disease, osteoporosis, diabetes mellitus, cancer, etc. Moreover, we have only investigated the adipogenic, osteogenic and chondrogenic differentiations but neural differentiation is still needed to be investigated as there is a relation between *MTHFR* C677T polymorphism and neural diseases.

In summary, this study demonstrates *MTHFR* C677T polymorphism affects adipogenic differentiation capacity and the clonality of the BM-MSCs. It is possible that there can be many other mutations that take place in the genome of the donors. Considering that every individual has a different genome, screening of donor cells with whole genome sequencing (WGS) or at least panels of known important mutations before cellular transplants can provide important information about the treatment effectiveness and efficiency, which must be supported with further studies. The success of the therapy and the future wellbeing of the patients are the final desire of both the clinicians and the patients. We believe that our study would contribute to the importance of possible effects of genetic mutations on the cells used for cellular therapies.

## Figures and Tables

**Table 1.1:** PCR primer sequences.

Primer name	Primer sequences (5′-3′)
**MTHFR-Forward**	**ACTCAGCGAACTCAGCACTC**
**MTHFR-Reverse**	**AGAGGACTCTCTCTGCCCAG**
**BNP-Forward**	**CAGCCTCGGACTTGGAAAC**
**BNP-Reverse**	**CTTCCAGACACCTGTGGGAC**

**Table 1.2:** PCR conditions for amplification.

Component	50 uL reaction
**5x My Taq Buffer (Bioline)**	10 uL
**MTHFR/BNP primers (F/R)**	2 uL
**My Taq DNA polymerase**	1 uL
**ddH** **_2_** **O**	Up to 50 uL
**100 ng DNA**	Volume determined depending on stock gDNA concentration

**Table 1.3:** Thermal cycler conditions of PCR.

	BNP	MTHFR
**Initial denaturation**	95 °C, 4min	95 °C, 4 min
**Denaturation**	95 °C, 30 s	95 °C, 30 s
**Annealing**	60 °C, 30 s	60 °C, 30 s
**Extension**	68 °C, 30 s	68 °C, 30 s
**Cycles**	35	35
**Final extension**	68 °C, 5 min	68 °C, 5 min

**Table 1.4:** RFLP conditions.

	For 50 uL of PCR product	
	Uncut	Cut
**CutSmart Buffer**	2.5 uL	2.5 uL
***Hinf1*** ** enzyme**	-	0.5 uL (U)
**ddH** **_2_** **0**	2.5 uL	2 uL

**Table 2:** BM-MSC marker expressions of the donors.

	CD73 (%)	CD90 (%)	CD105 (%)	HLA-DR (%)	CD34 (%)	CD45 (%)
**WT1**	99.8	99.9	99.8	0.1	0.2	0.1
**WT2**	99.9	99.9	99.7	0.1	0.1	0.1
**WT3**	99.8	99.7	99.9	0.1	0.1	0.1
**HTZ1**	99.7	99.3	99.7	0.2	0.1	0.1
**HTZ 2**	96.4	95.5	96.6	0.1	0.1	0.1
**HTZ 3**	99.8	99.4	99.5	0.3	0.7	0.1
**HMZ 1**	99.8	99.9	99.1	0.1	0.1	0.1
**HMZ 2**	99.7	98.0	99.5	0.1	0.1	0.2
**HMZ 3**	99.3	99.8	99.5	0.1	0.7	0.1
